# In Search of Cellular Immunophenotypes in the Blood of Children with Autism

**DOI:** 10.1371/journal.pone.0019299

**Published:** 2011-05-04

**Authors:** Paul Ashwood, Blythe A. Corbett, Aaron Kantor, Howard Schulman, Judy Van de Water, David G. Amaral

**Affiliations:** 1 Department of Medical Microbiology and Immunology, University of California Davis, Davis, California, United States of America; 2 The M.I.N.D. Institute, University of California Davis, Davis, California, United States of America; 3 Department of Psychiatry and Behavioral Sciences University of California Davis, Davis, California, United States of America; 4 PPD Biomarker Discovery Sciences, Menlo Park, California, United States of America; 5 Division of Rheumatology, Allergy and Clinical Immunology University of California Davis, Davis, California, United States of America; Chiba University Center for Forensic Mental Health, Japan

## Abstract

**Background:**

Autism is a neurodevelopmental disorder characterized by impairments in social behavior, communication difficulties and the occurrence of repetitive or stereotyped behaviors. There has been substantial evidence for dysregulation of the immune system in autism.

**Methods:**

We evaluated differences in the number and phenotype of circulating blood cells in young children with autism (n = 70) compared with age-matched controls (n = 35). Children with a confirmed diagnosis of autism (4–6 years of age) were further subdivided into low (IQ<68, n = 35) or high functioning (IQ≥68, n = 35) groups. Age- and gender-matched typically developing children constituted the control group. Six hundred and forty four primary and secondary variables, including cell counts and the abundance of cell surface antigens, were assessed using microvolume laser scanning cytometry.

**Results:**

There were multiple differences in immune cell populations between the autism and control groups. The absolute number of B cells per volume of blood was over 20% higher for children with autism and the absolute number of NK cells was about 40% higher. Neither of these variables showed significant difference between the low and high functioning autism groups. While the absolute number of T cells was not different across groups, a number of cellular activation markers, including HLA-DR and CD26 on T cells, and CD38 on B cells, were significantly higher in the autism group compared to controls.

**Conclusions:**

These results support previous findings that immune dysfunction may occur in some children with autism. Further evaluation of the nature of the dysfunction and how it may play a role in the etiology of autism or in facets of autism neuropathology and/or behavior are needed.

## Introduction

Autism is a lifelong neurodevelopmental disorder characterized by social deficits, impaired verbal and nonverbal communication and the presence of stereotyped behaviors or circumscribed interests [1]. Autism, together with Asperger syndrome and pervasive developmental disorder not otherwise specified, referred to as autism spectrum disorders (ASD), form a spectrum of conditions with varying degrees of impairment that are classified as pervasive developmental disorders in the DSM-IV [2]. The current estimate of prevalence is approximately 1∶110 [3], which is substantially higher than earlier estimates [4]. Numerous attempts at determining susceptibility genes through a number of large consortia have indicated that multiple genes, including immune related genes, may be associated with autism. Interestingly, none of the defined mutations, genetic syndromes and *de novo* copy number variations account for more than 1–2% of cases of autism [5].

There has been substantial speculation about the etiology(ies) of ASD, but for the vast majority of cases, the cause remains unknown. It has become clear that there will be many causes of autism that will likely have varying contributions from genetic and environmental factors. One persistent suggestion has been that an immune dysfunction may contribute to certain forms of autism. There have been numerous findings of altered immune function in autism [6]. As long as 45 years ago, Stubbs [7] noted that children with autism had altered responses to T cell mitogens, such as phytohemagglutinin or pokeweed antigen and these findings have been replicated in subsequent studies [8]–[10]. More definitive studies have since highlighted the presence of inflammation in the brain and the activation of microglia [11] as well as evidence for altered peripheral immune function in autism, including increased cytokine levels in the plasma such as interleukin (IL)-1β, IL-6, and IL-8 [12], elevated levels of complement proteins [13], decreased cellular activity of NK cells [14]–[16], increased monocyte activation [17], [18], and a reduced number of CD4+ T cells [10], [19], [20]. Pliopys et al. [21] reported that a substantial number of individuals with autism demonstrated an increased number of HLA-DR+ T cells and this finding has been confirmed by Warren et al. [22]. In addition, a number of studies have reported abnormal antibody responses to brain and CNS proteins [23], [24]. Skewed immunoglobulin (Ig) responses, such as decreased total serum IgG levels but increased isotype IgG4, have also been reported in autism [25]–[27].

Taken together, these data are suggestive of a link between autism and immune dysfunction and that specific cellular phenotypes or activation status of immune cells may be altered in autism. Autism is also associated with a variety of co-existing symptoms including seizures, sleep disturbances and gastrointestinal problems [28] many of which may be influenced by altered immune function. However, the data are often clouded by methodological concerns. The often heterogeneous populations of subjects analyzed, the use of siblings as controls and the disparate age ranges between controls and cases have led reviewers of this literature to be very cautious in drawing conclusions. Krause et al. [29] conclude that “Although various immune system abnormalities, involving both cellular and humoral aspects of the immune system, have been reported in children with autistic disorder, previous studies are largely association based, and, it remains difficult to draw conclusions regarding the role of immune factors in the etiopathogenesis of this neurodevelopmental disorder.”

The current study was designed to search for cellular markers of autism. Participants were selected from a very narrow age range (4 to 6 years) of children, to coincide with peak symptom presentation and to ensure a stable diagnosis. In addition, participants were only enrolled in the diagnostic group if they had a confirmed diagnosis of strictly defined autistic disorder (N = 70). Similarly, an age and gender matched control group of typically developing children (N = 35) was comprehensively evaluated to avoid inclusion of individuals with an autism spectrum or other neurodevelopmental disorder. The aim of the investigation was to evaluate changes in the frequency of distinct cellular phenotypes in autism with the goal of identifying immune-specific differences that could be further investigated for a potential role as biomarkers. A number of parameters of immune system status including cell number, cell ratios and cell surface antigen intensities were assessed using microvolume laser scanning cytometry.

## Methods

### Participants

The experimental subjects were recruited from the UC Davis M.I.N.D. Institute Clinic and community support groups such as Families for Early Autism Treatment (FEAT), Regional Centers, referrals from clinicians, and area school districts. Parents of children who met the diagnostic criteria were provided with an information sheet containing a description of the study and contact information. Typically developing subjects were recruited from area school districts and community centers. Informed written consent was obtained from parents prior to any assessments or procedures. The study was explained in simple language to the children and verbal assent was obtained from higher functioning children who were capable of understanding the study process. All participants were assigned a numerical code to maintain anonymity of the children and their test results. The subject number served as the primary identifier on research data forms. Following informed consent, interested subjects completed the diagnostic and psychological measures. All facets of the study were approved by the University of California Davis Institutional Review Board (IRB). For the duration of the study, there were no adverse events.

The inclusion criteria for the three groups consisted of the following. The children in the autism diagnostic group required a diagnosis of Autistic Disorder based on the DSM-IV criteria [2]. Children with pervasive developmental disorder-not otherwise specified (PDD-NOS) or Asperger Syndrome were excluded from the study. The diagnosis of Autistic Disorder was corroborated by: 1) the Autism Diagnostic Observation Schedule-Generic (ADOS-G; [30], 2) Autism Diagnostic Interview-Research (ADI-R; [31], [32], and 3) clinical judgment by one of the authors (BAC). The ADOS-G was used to assess children with autism to confirm diagnosis for inclusion in the study and is comprised of four different modules that are administered based on the language ability of the child. The ADOS provides an algorithm with cut-offs for autism and autism spectrum disorders [33]. The Autism Diagnostic Interview-Revised (ADI-R; [31] was administered to the parents of children with suspected autism. The ADI-R generates a diagnostic algorithm based on the DSM-IV [2] criteria for Autistic Disorder. The autism diagnostic group was further divided based on the level of intellectual functioning as follows: High functioning autism (HFA) having an IQ≥68 and low-functioning autism (LFA) having an IQ<68. The typically developing children (TYP) had intellectual functioning within the average to above average range with a minimum IQ≥68.

Typically developing control children were assessed using the Social Communication Questionnaire (SCQ; [34], a valuable screening tool that was completed by parents to ensure the absence of symptoms of autism. Children who had scores above the cutoff were excluded from the typically developing group, and were referred for further diagnostic evaluation (N = 1). In addition, all children were assessed using the Stanford-Binet Intelligence Scale-Fourth Edition [SB4; 35], a standardized measure of cognitive functioning for children between 2 and 18 years, which is divided into several parts, including Verbal Reasoning, Abstract/Visual Reasoning, Quantitative Reasoning, and Short-term Memory. The SB4 was used to obtain a measure of overall IQ for inclusion into the study as well as for the assignment to high (≥68) and low (<68) functioning autism groups. Similarly, all children underwent assessment with the Vineland Adaptive Behavior Scales-Interview Edition (VABS; [36], a structured parent interview format designed to assess a child's ability to perform daily activities required for personal and social sufficiency; this was administered to obtain a measure of adaptive functioning across all participants. As a further assessment, the AGRE Medical History Forms were given to the parents of all subjects to provide a comprehensive medical history. The interview requires the parent to provide demographic, medical and family history information.

The exclusion criteria for all subjects consisted of the presence of Fragile X or other serious neurological (e.g., seizures), psychiatric (e.g., bipolar disorder) or known medical conditions including autoimmune disease and inflammatory bowel diseases/celiac disease. All subjects were screened via parental interview for current and past physical illness. Children with known endocrine, cardiovascular, pulmonary, liver or kidney disease were excluded from enrollment in the study.

A total of 136 children between 4-years, 0-months and 6-years, 11-months were enrolled in this investigation. Twenty-one children were excluded due to failure to meet inclusion criteria or noncompliance with the majority of the protocol. The final sample consisted of 105 children who were selected to balance age and gender across experimental groups. Demographic information for the 105 subjects is presented in Table 1 including gender, age, IQ and ethnicity across the groups.

**Table 1:** Demographic Variables for the three groups of subjects evaluated in this study.

Characteristic	HFA	LFA	TYPICAL
N	35	35	35
Male∶Female Ratio	29∶6	29∶6	29∶6
Age (Median)	5.2	5.5	5.7
IQ	79	56	115
Caucasian	23	21	30
Hispanic	5	6	0
Asian	3	2	2
African-American	0	1	1
Other	4	5	2
ADOS total (mean ± SD)	13.3±2.2	15.9±2.4	-
ADOS communication (mean ± SD)	5.5±1	5.8±1.4	-
ADOS social (mean ± SD)	7.8±1.8	10±1.9	-
ADOS play (mean ± SD)	1.3±1.2	3.3±1.1	-
ADOS repetitive (mean ± SD)	2.3±1.1	3.8±1.1	-
ADI social (mean ± SD)	18.3±6.7	24.9±3.2	-
ADI verbal (mean ± SD)	14.5±4.7	16.4±2.9	-
ADI nonverbal (mean ± SD)	7.8±4.9	12.9±1.5	-
ADI repetitive behavior (mean ± SD)	6.8±2.8	5.8±1.7	-
ADI Abnormal behavior (mean ± SD)	4.1±0.9	4.7±0.5	-

Participation in the study required two visits. Child assessments and parental interviews were conducted at the UC Davis M.I.N.D. Institute Research Clinic on the first visit; testing lasted for approximately 3 1/2 contact hours. The parents were sent letters providing the results of their child's performance. The second visit consisted of a blood draw completed by a pediatric phlebotomist and research staff via standardized procedures (see below).

### Sample Collection Procedures

For each child, approximately 5 ml of blood was drawn by one of two clinical phlebotomists into Vacutainer tubes containing EDTA (BD Biosciences, San Jose, CA). Immediately following collection, the tube was gently inverted 8 to 10 times to mix the anticoagulant with the blood. The tube was then wrapped in parafilm and bubble wrap and placed in a biohazard bag between coolant packs in a Styrofoam transport container. The blood draws were taken within one week of the diagnostic and psychological assessments for each of the children. All blood draws were conducted in the early morning hours between 8:00 am and 10:00 am following an overnight fast (no consumption of food or drink other than water after midnight). Topical anesthetics were not employed to prevent contamination of the sample. If the participant was ill (presented with a cold, fever or other common illness), the blood draw was not taken until the child's health status was stable/recovered for 48 hours. The samples were sent via Courier to PPD Biomarker Discovery Sciences (Menlo Park, CA, USA, formerly known as SurroMed, Inc.) arriving in the lab within six hours of the blood draw. Cytometric analyses of the blood samples were carried out immediately on receipt of the samples in the lab. PPD personnel were blind to the diagnosis until after all samples were assayed and a report of preliminary findings was presented.

### Analytic Methods

The protocol for immune phenotyping included 64 three-color cellular assays performed by microvolume laser scanning cytometry on the SurroScan™ system [37]–[39]. The assays are well-suited for evaluating cellular immune markers. Monoclonal antigen-specific antibodies were purchased from various commercial vendors and developed into PPD assays. Three different fluorophores, Cy5, Cy5.5 [40], [41] and the tandem dye Cy7-APC [42], [43], were coupled to individual monoclonal antibodies specific for different cellular antigens in each assay. Each fluorophore was measured in a separate detection channel. Aliquots of whole blood were added to 96-well micro-titer plates containing the appropriate antibody-dye combinations for each assay, incubated in the dark at room temperature for 20 minutes, diluted with an appropriate buffer and loaded into Flex32™ capillary arrays (PPD) and analyzed with SurroScan™. Images were converted to a list-mode data format with in-house software [44]. Fluorescence intensities were compensated for spectral overlap of the dyes so values are proportional to cell surface antigen density. Standard beads were run with every sample and were used to monitor systematic instrument errors.

Prior to this study, PPD developed and established quality and baseline measures with twenty blood bank samples for the 64 different three-reagent cellular assays used in this study. Standard template gates were established using these results plus additional staining controls for all individual reagent and dye combinations. Template gates were established using FlowJo™ cytometry analysis software (Tree Star, Inc., Ashland, OR) customized for PPD to enable upload of gates to an Oracle database. Gating information was stored in the database and applied to the scan data for each assay using SurroGate™ database-driven cytometry analysis software in order to generate the resulting cell count and antigen intensity data.

The assay panel allows the enumeration of major cell populations: granulocytes, eosinophils, monocytes, CD4 and CD8 T cells, B cells and NK cells. In addition, the assays allow for finer phenotyping of cell subtypes based on the expression of specific cell surface markers of activation, adhesion molecules, receptors, etc. The assays monitor cell counts of more than 200 different cell populations, plus the relative levels of the different cell surface antigens on specific populations. Template gates were used to enumerate the cell populations of interest in all of the assays. Invalid assays and those that do not support the template gates were flagged. An analyst visually reviewed all assay results prior to data upload. In this study, 105 subject samples were analyzed with 64 assays for a total 6720 assays. Among the assays, only 0.67% were invalid due to technical difficulties and have been excluded from the analysis. An additional 4.8% required non-standard gates due to slight inter-individual differences. These results are used in the statistical analysis. Cell counts were generally not affected but cell surface expression results may have a larger but not statistically significant variation due to the inclusion of these data.

### Statistical Analyses

Statistical analyses were conducted to assess differences in cell populations, 1) between the combined autistic group (HFA+LFA) and the control group, 2) between each of the autism subgroups and 3) among the three groups. With regard to two-group comparison statistics, we applied to all data a univariate mean comparison test that was either parametric or non-parametric depending on the normality of the data. If the data were approximately normally distributed, then parametric statistics were used (t-test); if not, the nonparametric rank test (Wilcoxon or Kruskal-Wallis test) was applied. All tests of hypotheses were two-sided. Goodness-of-fit statistics (Shapiro-Wilk) and tests of skewness and kurtosis were performed to assess normality. Three-group comparisons were performed by ANOVA. The data set for this study is broad, i.e., there are many more variables than subjects. Consequently, many multivariate statistics such as multivariate analysis of variance, which require more subjects than variables, could not be conducted. This study was underpowered for the number of variables being studied and some interesting results would be overlooked if the univariate statistics were ignored. Although the groups were carefully controlled and matched for sex and ethnicity, the study is underpowered to find differences based on sex and ethnicity. Unadjusted P values are presented since this study is preliminary and is the first to begin to explore cellular markers on different blood cells. Moreover, the use of correction for multiple comparisons in this area is debated [45]. Our hypothesis tests included 644 variables from cell counts and cell surface marker intensities. Multiple measures of the same cell population (e.g. CD4 T cells) were combined into a single average for the analysis. Differences at the univariate p-value<0.05 or lower, warrant further consideration.

## Results

There were multiple significant differences observed in immune cell numbers and the surface expression of markers on immune cells in children with autism compared with age and gender-matched typically developing controls. For example, at the p<0.05 level there were 151 variables where either cell count or the intensity of cell surface markers were different between children with autism and controls (Table 2).

**Table 2:** Significantly different immunophenotyping measures (including both cell counts and fluorescence intensities) between study groups.

P value criteria	Chance	Autism vs. Typically developing	HFA vs. Typically developing	LFA vs. Typically developing	HFA vs LFA	HFA vs. LFA vs. Typically developing
p<0.001	<1	21	3	22	1	12
p<0.01	6	77	25	76	7	54
p<0.05	32	151	101	162	33	139

Based on 644 variables.

In general, more differences were observed between children with autism and typically developing controls than between the low functioning (LFA) and high functioning (HFA) autism groups based on IQ, although 33 variables were different between HFA and LFA at the p<0.05 level. A summary of the significant measures for each of the comparisons is shown in Table 2. For each statistical level (p-value) the number of false-positive variables expected to appear by chance (assuming all are independent) is given in the first column. The significant differences between autism and controls included both differences in cell counts and, separately, differences in the intensity of cell surface marker expression. Tables 3 and 4 represent variables that were significantly different for cell counts and cell surface expression markers intensities, respectively.

**Table 3:** Significant measures for study comparisons - counts only.

P value criteria	Chance	Autism vs. Typically developing	HFA vs. Typically developing	LFA vs. Typically developing	HFA vs LFA	HFA vs. LFA vs. Typically developing
p<0.001	<1	5	1	2	0	2
p<0.01	2	23	11	14	2	10
p<0.05	11	43	42	39	4	36

Based on 224 count variables.

**Table 4:** Significant measures for study comparisons - intensity only.

P value criteria	Chance	Autism vs. Typically developing	HFA vs. Typically developing	LFA vs. Typically developing	HFA vs LFA	HFA vs. LFA vs. Typically developing
p<0.001	<1	16	2	20	1	10
p<0.01	4	54	14	62	5	44
p<0.05	21	108	59	123	29	103

Based on 420 fluorescence intensity variables.

### Analysis of immune cell counts

Cell count data were available for the major immune cell populations i.e., neutrophils, lymphocytes, eosinophils, monocytes and platelets. We found that the absolute numbers (cells per microliter) of B cells and NK cells in children with autism were significantly higher than counts from typically developing controls. Although there were higher mean absolute numbers of total white blood cells (WBC), neutrophils, T cells, the CD4 and CD8 T cell subpopulations, monocytes, eosinophils and platelets in children with autism, these differences in cell counts did not reach statistical significance (Table 5).

**Table 5:** Comparison of major blood cell populations between Autism and Typical groups.

		Typical Developing controls (N = 35)		Autism (N = 70)		P-Values
Cell Population	Trend	Mean	SD	Mean	SD	
WBC	↑	7524	1783	8220	2238	0.169
Granulocytes	-	3441	1474	3582	1519	0.557
Neutrophils	-	3398	1490	3401	1435	0.747
T cells	-	1834	629	1961	612	0.330
CD4 T cells	-	1118	437	1211	459	0.330
CD8 T cells	-	664	267	686	249	0.560
B cells	↑	542	279	661	255	0.003
NK cells	↑	117	80	161	95	0.011
Monocytes	-	446	182	453	188	1
Eosinophils	↑	286	225	438	515	0.066
Platelets	↑	1430357	505854	1613025	628482	0.188

N is the number of subjects. Values are absolute cell numbers per microliter. Univariate p-values are shown.

### Analysis of B cells

**Figure 1:**
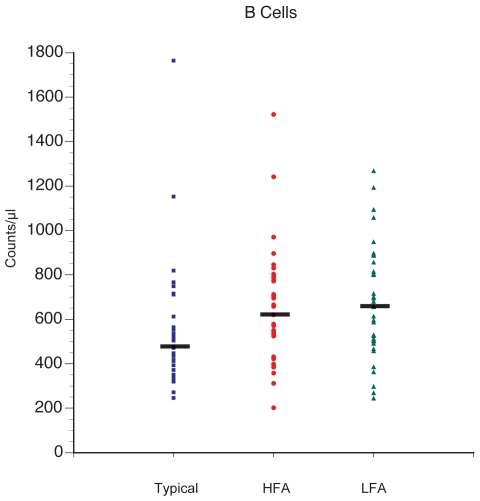
Average B cell counts are higher in the HFA and LFA groups compared with controls. The absolute count values are based on 9 separate B cell assays that use CD20 as the B cell identifier. P-values = * HFA vs. N = 0.011, ** LFA vs. N = 0.009, and A vs. N 0.003. HFA vs. LFA = not significant (0.7).

Absolute numbers of B cells were 20 to 25% higher in the autism groups compared with the typically developing controls. The B cell value represents an average based on nine separate B cell assays that use CD20 as the B cell identifier. No differences were seen within the autism group when comparing HFA with LFA. B cell counts were significantly higher for both LFA (p = 0.009) and HFA (p = 0.011) after adjustment for multiple comparison compared with typically developing controls (Figure 1). In addition, there were significant differences in activated B cell subsets, including statistically significant increases in B cells that expressed the activation marker CD38 in autism compared with controls (394.7±119.9 vs. 479.7±210.2, p = 0.0081, Table 6). The difference in CD38 positive (CD38p) B cell number in autism tracked with the increase in total B cell population. CD38 negative (CD38n) cell numbers were also higher but to a lesser extent (164.5±99.3 vs. 193.4±84.6, p = 0.013). There were also increases in mature B cell numbers as denoted by the absence of CD5 staining (CD5n) on B cells in autism subjects compared with typically developing controls (Table 6, p = 0.0001). Notably, although the number of B cells was increased in autism, the number of immature B cells, as denoted by positive CD5 staining, was not different between autism and controls (278.3±142.1 vs. 329.4±171.8, p = 0.14). These data suggest that B cells are increased in autism and it is preferentially the activated and mature phenotype which differs from controls.

**Table 6:** Comparison of B cell subsets.

		Typical Developing controls (N = 35)		Autism (N = 70)		P-Values
Cell Population	Trend	Mean	SD	Mean	SD	Univariate
B cells	↑	542	279	661	255	0.003
CD5n	↑	268.26	167.45	348.89	139	0.001
CD38p	↑	394.65	199.91	479.71	210.22	0.008

N is the number of subjects. Values are cell numbers per microliter. Univariate p-values are shown.

### Analysis of NK cells

**Figure 2:**
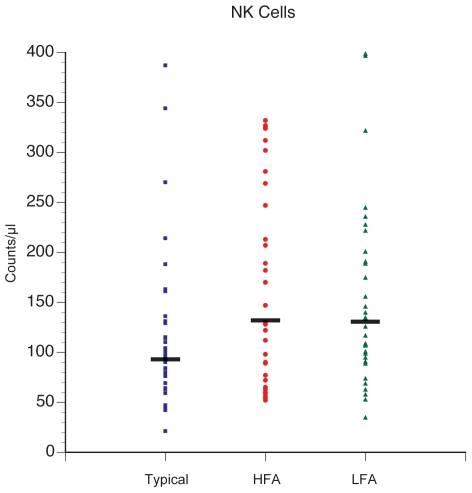
Average NK cell counts are higher in the HFA and LFA groups compared with typically developing controls. The average is based on 2 separate NK cell assays that use the CD56p and CD3n as the NK cell identifier. P-values = *HFA vs. N = 0.037, *LFA vs. N = 0.023, and A vs. N = 0.011. HFA vs. LFA = not significant (0.8).

Absolute numbers for NK cells were approximately 40% higher in children with autism compared with controls (Table 5). The measure of NK cells was based on an average of two separate NK cell assays that use the markers CD56 and CD3, so that NK cells are identified as CD56pCD3n. The difference in NK cell numbers was significant for both HFA vs. controls (p = 0.037) and LFA vs controls (p = 0.023), but no differences were observed between HFA and LFA (Figure 2).

### Analysis of cell surface marker intensities

As indicated in Table 4, there were many significant differences in the intensity of cell surface markers expressed on immune cells. At the p<0.05 level, there were 108 different variables denoting intensities that were different between children with autism and controls (Table 4). To help organize the data that are based on cell surface marker intensities, we reviewed the top 20 intensity variables with differences greater than 15% between the autism and control groups and had adjusted p-values<0.05. These variables are listed in Table 7. It is important to note that a number of these variables represent cell surface markers that are only present on rare cells for which the observed cell counts were very low.

**Table 7:** The 20 most different cell surface markers based on fluorescent intensities.

Assay panel	Cell surface marker	Trend	Autism (N = 70)		Typical Developing controls (N = 35)		P-Value	% Ratio
			Mean	SD	Mean	SD		
**Intensity Differences >31%**								
CD16pCD66bpCD52n	CD52	↑	4453.5	1399	3395.8	1622.2	0.000	131
CD4pnCD14pCD95p	CD95	↑	3632.8	1629.4	2764.9	705.8	0.002	131
CD3pCD4nHLADRp	HLA-DR	↑	2746	2019.5	2073.8	1281.9	0.020	132
CD4pnCD14pCD25p	CD4	↑	254.2	189.5	193.6	196	0.040	131
**Intensity Differences 25–30%**								
CD7pCD8pCD26p	CD26	↑	1149	390.5	887.4	202.4	0.000	129
Neutrophil-CD66b	CD66b	↑	4574.8	1275.6	3531.8	1310.6	0.000	130
CD16pCD66bpCD52p	CD66b	↑	4415	1392.3	3489	1284.5	0.002	127
CD8pnCD57pCD94p	CD94	↑	1911.4	1016.2	1473.3	780.4	0.040	130
**Intensity Differences 21–25%**								
CCR5nCD8pCD60n	CD8	↑	2278.5	479.4	1873.8	451.7	0.000	122
CD8pCD20nCD38n	CD8	↑	2762.6	691	2285.8	612.5	0.001	121
CD3pCD4pHLADRp	HLA-DR	↑	1166.5	626.2	964.7	991	0.001	121
CD8nCD16pCD101p	CD101	↓	970.5	439.5	1228	383.2	0.004	79
CD11bpnCD16pnCD32p	CD32	↓	745.7	573.1	964.1	685.6	0.030	77
CCR5pCD4pCD60p	CD4	↑	1594.7	920.9	1282.4	638.7	0.030	124
**Intensity Differences 16–20%**								
CD8pCD45RApCD60p	CD8	↑	2717.3	462.1	2343.4	463.6	0.000	116
CD8pCD20nCD95n	CD8	↑	2306	472.2	1981.9	404.1	0.001	116
CD11bpCD16p	CD11b	↑	2865.7	661.6	2390	691	0.001	120
CD14nCD15pCD89p	CD15	↑	2478.2	619.4	2112.4	722	0.006	117
CD16pnCD18pCD44p	CD44	↓	1654.1	740.3	2004	808.5	0.012	83
CCR5pCD8pCD60n	CCR5	↑	856.6	324.1	739.8	232.6	0.030	116

Abbreviations: p = positive staining, n = negative staining, pn = weak positive staining, N = number. Univariate P value is shown to 3 decimal points only.

Ranking is based on intensity differences of 15% or more between autism and typically developing controls and statistical significance.

Of interest, HLA-DR, a marker of cellular activation, was higher on CD8 T cells and CD4 T cells in the autism group compared with typically developing controls. The T cell marker CD26/dipeptidyl peptidase IV, which is associated with an effector cell phenotype and is markedly elevated in human CNS disorders such as multiple sclerosis, was increased on CD8 T cells in autism compared with controls. Another noteworthy finding was that CD95 expression was increased on CD14 expressing monocytes compared with controls. The marker CD95 is often expressed on activated cells as a means of making those cells more susceptible to apoptosis in order to limit the inflammatory response. Increased CD95 on monocytes from children with autism may represent an activated subset of monocytes that have upregulated the surface expression of this apoptosis marker.

## Discussion

The current study was designed to search for cellular markers of autism. Given the genetic heterogeneity of autism [5] and the near certainty that autism spectrum disorders have many etiologies and trajectories, it is noteworthy that the current study has identified several indications of immune differences in children with autism. In general, we found that the frequencies and phenotypes of whole blood immune cell subpopulations under non-stimulated conditions were different in children with autism compared with well matched, typically developing controls. Based on 644 measurements relating cell counts of immune subsets and the abundance of cellular markers, as determined by the intensity of antibody staining directed to these markers, nearly a quarter (151) of these measurements were different between children with autism compared with controls at the univariate p<0.05 statistical level. There was additional evidence of differences between higher functioning autism participants and lower functioning autism participants with 33 of the measured variables being different between the autism groups at the p<0.05 level. Notably, the data highlighted significantly higher absolute numbers of B cells and NK cells in children with autism compared with controls. In addition, increased markers of cellular activation, such as CD38 on B cells, and HLA-DR and CD26 on T cell subsets, were observed on cells from autism participants compared with controls.

Previous reports have demonstrated differences in lymphocyte populations in autism, including increased numbers of NK cells [14] and reduced numbers of T cells [10], [19], [20], [46], [47] or altered activation status of T cell subsets [8], [21]. In the current study, participants were selected in a very narrow age range (4-to-6 years), to coincide with peak symptom presentation and to ensure a stable diagnosis; the male to female frequency was the same in each group (29 males to 6 females). Participants in the diagnostic group had strictly defined autistic disorder and were compared to a comprehensively evaluated control group of typically developing children, none of whom were siblings of the autism cases. It is difficult to make direct comparisons between our study and the majority of previous studies due to differences in analytical technique, the age range of the subjects, the diagnostic criteria used, the lack of confirmation of the absence of ASD or other neurodevelopmental disorders in the controls and the use of siblings as controls. In addition, in previous studies there may also have been unintentional selection bias due to recruitment through specialist clinics that may have skewed selection of only children with regression or children with overt gastrointestinal symptoms. However, taken together, the current study and existing literature would strongly support the hypothesis that cellular immune abnormalities exist in a substantial subset of children with autism.

Findings from our current study describe increases in NK cells from children with autism compared to typically developing controls. These data are in line with a previous report of greater frequencies of NK cells and increased gene expression of NK cell-related cellular receptors and effector molecules in children with autism [14]. A number of studies have however, shown decreased responsiveness of NK cells to *in vitro* stimulation [14]–[16]. The increase in NK cell numbers seen in this study may therefore reflect a compensatory mechanism to increase cell numbers to make up for possible deficits in NK cell function. However, reduced activation after stimulation could also occur if the NK cells were already maximally stimulated *in vivo*, a phenomenon frequently observed in autoimmune diseases. Furthermore, NK cells have been shown to play a critical role in the initiation of autoimmune-like responses in diabetes and celiac disease [reviewed in 48]. The increased presence of auto-antibodies to brain and CNS proteins is a common finding in autism and may reflect an ongoing inflammatory and or autoimmune process in children with autism that could be initiated by abnormal NK cell activation [6]. In this case, the expansion of NK cell numbers may result from heightened immune/autoimmune responses most likely mediated through the increased production of homeostatic and growth factors such as cytokines.

The frequency of mature (CD5n) and activated (CD38p) B cells were also increased in this study and could also contribute to increased production of auto-antibodies. Cytokines such as IL-6 participate in the activation and differentiation of B cells and their production of antibodies; previous studies have shown that there are increased plasma IL-6 levels in children with autism which could modulate B cell activity [12]. In addition, T cells help B cells to produce antibodies typically through the production of cytokines. Recent studies show that *in vitro* stimulation of T cells leads to increased production of IL-13 and IL-5 that promote activation and antibody production from B cells [8], [9]. Moreover, in this study we find that T cells express a profile of cell surface markers such as HLA-DR and CD26 that are indicative of activation and are in line with previous reports of altered T cell activation in children with autism [8], [22]. Taken together, our results suggests that there is an activation of immune responses in children with autism that leads to increased frequency of NK cells, and activated B cells and T cells. It is tempting to further suggest that these cell types may interact in such a way as to break immunological tolerance to self proteins and to elicit auto-immune responses leading to the production of auto-antibodies. The balance between regulatory T cells and T_H_17 cells are important in the initiation of autoimmune diseases. In our previous studies, we did not find a difference in the frequency of circulating FoxP3^+^ or CD25^++^ regulatory T cells under resting conditions [8], [49]; however, regulatory cell function in children with autism may be altered as we have shown decreases in TGFβ1 levels [50] and IL-10 production [51], [52]. So far, in children with autism aged between 2 and 5 years of age we have found no differences in IL-17 plasma levels [53], T_H_17 cell frequency at baseline levels or following stimulation, and IL-17 production following stimulation [49]. Taken together these data do not suggest that there are differences in T_H_17 in children with autism of this age range but they can not rule out earlier alterations in T_H_17 cell function that may be linked to causation of autism. Further assessments of T_H_17 and regulatory T cells and their interactions in autism needs further investigation.

We, and others, have performed preliminary analyses that suggest that certain immunological parameters are associated with specific behavioral symptoms in autism. Impairments in social behaviors, for example, are associated with decreased levels of TGFβ1 [50], increased IL-1β and IL-13 [12], increased macrophage inhibitory factor [54], decreased platelet-endothelial adhesion molecule [55], total IgG [27], increased IgG4 isotype [26], altered T cell responses [8], chemokine levels [56] and activated monocyte responses [17]. In addition, parallel proteomic analysis of sera samples from the same participants in this study showed increased immune profiles with marked differences in complement components in children with autism compared with typical developing controls [13]. The causal link, however, between these immune factors and behavioral output remains unexplored. Moreover, no one marker is diagnostic of autism and the results reported here imply that panels or “signature” profiles containing multiple markers may associate more specifically with distinct symptomatology. Further work to determine the potential use of immune based panels as the basis for defining autism phenotypes is called for. These studies should include “at-risk” groups where identification of autism before behavioral symptoms are manifest would be a major advance for the care and management of such individuals. However, a major challenge in the identification of reliable early biomarkers is to minimize the effects of confounding factors such as medication, which may be more prevalent in the autism group. In this study, we recruited drug-naïve participants, and carefully screened and selected well-matched controls. Future studies will need to consider collecting even more in-depth and extensive demographic information about all aspects of lifestyle in order to minimize the effects of as yet unidentified potential confounders.

Currently there is insufficient evidence to refute or confirm the presence of specific immune dysfunction in autism and it is still unclear whether immune alterations are reflective of specific immune dysfunction, or a bystander effect of upstream regulatory mechanisms, or result from tissue pathology associated with the disorder. This study does not seek to confirm or address specific immunological issues in children with autism but rather to investigate whether immune parameters may be useful as biological markers in autism. To start to address the complexity of this symptomatically defined disorder, we need to conduct studies aimed at identifying clear and consistent differences between individuals with autism and controls. One potential limitation of the current study is that it was cross-sectional and that we only looked at the immune parameters at one time point. Immune responses are exceedingly dynamic and it is often hard to achieve a comprehensive assessment of an immune response by looking at just one point in time. Also, physiological inflammation may be transient and could be missed if participants are sampled at one time point only. Larger immunological studies need to be carried out, where several parameters can be measured and correlated for the same individual with follow up tests throughout the progression of the disorder.

A major problem of studies of autism to date has been the inability of one study to replicate the biological markers found in another study. This applies to almost any aspect or facet measured in subjects with autism. As autism is a complex and heterogeneous disorder that may have multiple causes, pathologies and trajectories, the assumption that autism is a single disorder, and that a given finding should extend across all subjects with autism, may not be tenable. In fact, one of the many difficulties in understanding autism may be that many different abnormalities may converge to produce similar behavioral symptoms in autism subjects. In order to begin to untangle the interaction between complex genetic and/or environmental factors in autism, it is essential to identify a reliable biomarker(s) that will help provide invaluable insight to elucidate mechanisms of action that underlie the causes of autism. Identifying these biomarkers could help identify different subgroups within the autism population. If possible, the discovery of true biomarkers will almost certainly yield more successful genetic and functional studies and ultimately will help in the design of efficacious treatments for autism.

A lack of objective, biomedical analytical tools is a serious limitation to the diagnosis of autism. There are several reasons to adopt multiplex immunoassays, including those presented in this study, as a technology to determine possible biomarkers in autism. “Signature” profiles of biomarkers that provide information regarding possible subclasses within autism and that correlate with behavioral states of autism would be extremely informative. An important aspect for future clinical application of such technology is that the potential biological signatures are suited to analyzing serial samples in longitudinal studies. The search for the identification of autism markers in the laboratory is an important research endeavor, yet the translation of such findings into the clinic is the real challenge and requires the investigation of much larger sample cohorts, ideally collected in different clinical centers. Further studies to examine the clinical power and utility of putative markers for autism, including those related to the immune response, are needed to help determine the usefulness in assisting early diagnosis of autism.
